# Carotid Stump Syndrome: Case Report and Endovascular Treatment

**DOI:** 10.1177/2324709614548796

**Published:** 2014-09-02

**Authors:** Lara Toufic Dakhoul, Rabih Tawk

**Affiliations:** 1 Advocate Christ Medical Center, Oak Lawn, IL, USA; 2 Mayo Clinic Hospital, Jacksonville, FL, USA

**Keywords:** neurosurgery, carotid stump syndrome, endovascular

## Abstract

*Objectives*. To highlight the case of a patient with multiple transient ischemic attacks and visual disturbances diagnosed with carotid stump syndrome and managed with endovascular approach. *Case Presentation*. We present the case of a carotid stump syndrome in an elderly patient found to have moderate left internal carotid artery stenosis in response to an advertisement for carotid screening. After a medical therapeutic approach and a close follow-up, transient ischemic attacks recurred. Computed tomographic angiography showed an occlusion of the left internal carotid artery and the presence of moderate stenosis in the right internal carotid artery, which was treated by endovascular stenting and balloon insertion. One month later, the patient presented with visual disturbances due to the left carotid stump and severe stenosis of the left external carotid artery that was reapproached by endovascular stenting. *Conclusion*. Considerations should be given to the carotid stump syndrome as a source of emboli for ischemic strokes, and vascular assessment could be used to detect and treat this syndrome.

## Introduction

Carotid stump syndrome (CSS) is one of the recognized causes of recurrent ipsilateral cerebrovascular events caused by occlusion of the internal carotid artery (ICA).^1^ As this infrequently occurs, it is responsible for ischemic strokes of the carotid territory. Microembolization from an ipsilateral ICA stump has been observed in the pathogenesis of such transient ischemic attacks (TIAs).^2^ The main pathophysiology of the syndrome is caused by the migration of microemboli to the ipsilateral external carotid artery (ECA) and the ophthalmic artery. These emboli move from the external carotid and ophthalmic arteries to the intracranial circulation via retrograde collateral pathways.

The article presents a case of a female patient initially suffering from left ICA occlusion that is diagnosed with CSS and managed with endovascular wall stenting.

## Case Presentation

An elderly patient was incidentally found to have moderate plaque in the left ICA after being screened by a carotid ultrasound on July 10, 2009. Three weeks later (July 29, 2009), duplex ultrasound was repeated; it showed left ICA occlusion with 50% to 79% stenosis of the right ICA. In fact, the patient had a history of long-standing hypertension, hyperlipidemia, and borderline diabetes mellitus; though taking a low dose of aspirin, she had a recurrence of TIAs episodes characterized by motor aphasia and speech changes.

Computed tomographic angiography confirmed the previous findings; while magnetic resonance angiography showed ulceration of the right stenosis, and so the patient’s aspirin dose was increased to 325 mg daily. Diagnostic cerebral angiogram with possible stenting of the right ICA was next taken. It showed a highly ulcerated stenosis of 60% at the bifurcation (Supplementary Figure 1), a left ICA occlusion (Supplementary Figure 2) and a left ECA stenosis. After inserting an embolic protection device, an 8 × 29 was deployed across the stenosis and a poststenting angioplasty was performed using a 4 × 20 mm balloon.

One month later (November 2, 2010), the patient had a 15-minute episode of seeing distortion with crooked and wavy lines; whereas, a fundoscopic examination revealed cholesterol emboli on the left retina (Supplementary Figure 3). The anatomic finding of the case indicated that a left carotid stump could be the source of the patient’s emboli.

Angiography, however, showed ICA occlusion with persistence of a carotid stump and severe stenosis of the left external carotid artery measuring 85%. With these findings, it was decided to proceed with angioplasty and stenting of the ECA using an 8 × 21 mm Boston Scientific wall stent across the stenosis and perform a poststenting angioplasty with a 3.5 mm aviator balloon (Supplementary Figure 4). After 6 months (January 4, 2011), a follow-up with computed tomography perfusion (with and without diamox) showed a normal bilateral blood flow in the brain without steal phenomenon, and the patient was asymptomatic with no neurological sequelae.

## Discussion

Carotid stump syndrome has been considered a rare cause of cerebrovascular diseases with many diagnosis and management problems. Its initial symptomatology includes TIA, cerebrovascular accident (with motor and sensory deficit), dysphasia, amaurosis fugax and visual loss. Though, the presentation indicates a difference between one patient and another due to the occluded territory.

Previous case reports showed a quite different post operative result ranging from an asymptomatic state (most cases) to an ipsilateral deterioration, mainly in visual acuity with a retinal infarct, to death several weeks postoperatively following an unrelated surgical procedure. The hemodynamic failure in the critical carotid axis led to cerebral hypoperfusion and cerebral ischemic symptoms.^3^ Two ways of management had been described in sporadic case reports: the endovascular technique and the surgical intervention. The endovascular technique mainly consisted of wall stenting across the stenosis (as in our patient) and/or poststenting balloon angioplasty. It was an interesting and effective alternative and a minimally invasive approach to management.^1^ However, the surgical intervention took into consideration the associated morbidity factors of the patients (risk vs benefit) but was not always a good choice.

A close control of the risk factors of thromboembolic events has also shown a significant influence on the recurrence of symptoms after the performance of the procedure.

Further investigations with more patients, additional case reports and control are done to achieve a better understanding and improve the results in patients with unilateral ICA occlusion and contralateral ICA stenosis.

## Conclusion

Transient ischemic attacks following ICA occlusion are not uncommon. CSS should be considered as a likely clinical entity in patients with an occluded ICA and persisting cerebral and retinal microembolic symptoms.^6^ The obstructing thrombus in a totally occluded ICA may act as a source of cerebral emboli. Endovascular treatment of CSS, with a concomitant well-directed medical therapy, has proved to play a role in improving the outcome of the patients with minimal morbidity. Therefore, a knowledge of the timing between the symptomatology from cerebral or retinal embolic events and ICA occlusion is crucial to diagnosis, management, and prognosis.^6^
